# The Cytokine IL-1β and Piperine Complex Surveyed by Experimental and Computational Molecular Biophysics

**DOI:** 10.3390/biom10091337

**Published:** 2020-09-18

**Authors:** Gabriel Zazeri, Ana Paula Ribeiro Povinelli, Marcelo de Freitas Lima, Marinônio Lopes Cornélio

**Affiliations:** 1 Departamento de Física, Instituto de Biociências, Letras e Ciências Exatas (IBILCE), UNESP, Rua Cristovão Colombo 2265, São José do Rio Preto CEP 15054-000, Brazil; gabriel.zazeri@unesp.br (G.Z.); ana.povinelli@unesp.br (A.P.R.P.); 2 Departamento de Química, Instituto de Biociências, Letras e Ciências Exatas (IBILCE), UNESP, Rua Cristovão Colombo 2265, São José do Rio Preto CEP 15054-000, Brazil; marcelo.f.lima@unesp.br

**Keywords:** cytokine, IL-1β, piperine, fluorescence spectroscopy, molecular docking, umbrella sampling

## Abstract

The bioactive piperine, a compound found in some pepper species, has been widely studied because of its therapeutic properties that include the inhibition of an important inflammation pathway triggered by interleukin-1 beta (IL-1β). However, investigation into the molecular interactions between IL-1β and piperine is not reported in the literature. Here, we present for the first time the characterisation of the complex formed by IL-1β and piperine through experimental and computational molecular biophysical analyses. Fluorescence spectroscopy unveiled the presence of one binding site for piperine with an affinity constant of 14.3 × 10^4^ M^−1^ at 298 K. The thermodynamic analysis indicated that the interaction with IL-1β was spontaneous (∆G = −25 kJ/mol) and, when split into enthalpic and entropic contributions, the latter was more significant. Circular dichroism spectroscopy showed that piperine did not affect IL-1β secondary structure (~2%) and therefore its stability. The set of experimental data parameterized the computational biophysical approach. Through molecular docking, the binding site micro-environment was revealed to be composed mostly by non-polar amino acids. Furthermore, molecular dynamics, along with umbrella sampling, are in agreement with the thermodynamic parameters obtained by fluorescence assays and showed that large protein movements are not present in IL-1β, corroborating the circular dichroism data.

## 1. Introduction

Interleukin-1β (IL-1β) is a protein that belongs to the cytokine family of cell mediators produced mainly by mononuclear phagocytes, activated in response to infection and injury [1]. With 17 kDa of molecular weight and 153 amino acids, this protein is the main soluble mediator of inflammation that triggers the activation pathway of the NF-κB responsible for the transcription of cytokine genes and other pro-inflammatory proteins [2]. The search for molecules with the potential to inhibit IL-1β function and consequently prevent the inflammation process is of utmost interest.

Ying et al. [2] reported that piperine inhibited the IL-1β-mediated activation of NF-κB and as consequence, the production of PGE2 and NO is downregulated as well as the IL-1β-stimulated gene expression and production of MMP-3, MMP-13, iNOS and COX-2 in human osteoarthritis chondrocyte. In another study, Bang et al. [3] showed that piperine acts through the inhibition of IL-1β and NF-κB inflammation pathway, leading to the downregulation of pro-inflammatory proteins, which reinforces the anti-inflammatory activity of this molecule.

**Figure 1:**
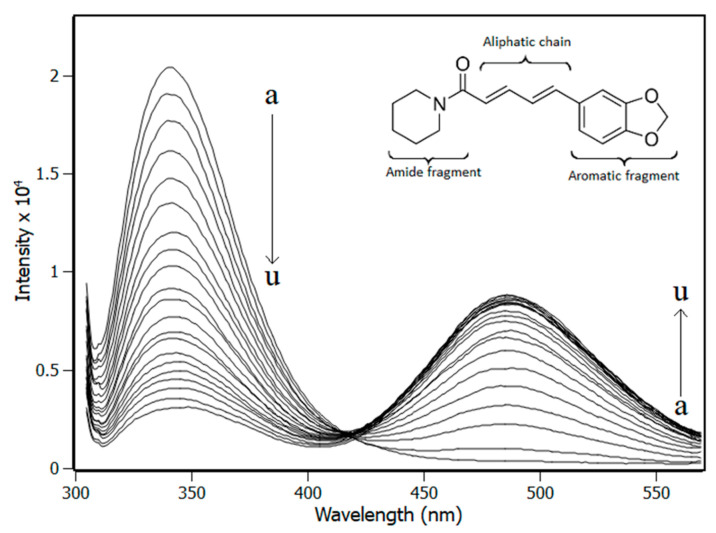
Spectra of fluorescence emission of IL-1β obtained from titration experiments with increments in the concentration of piperine (pH 7.4, T = 298 K, λ_ex_ = 295 nm). [IL-1β] = 4.0 μM; Piperine titrations with increment of 4 μM (a → u). The inset is a representation of the molecular structure of piperine.

Piperine (Figure 1) is an alkaloid found in some piper species such as *Piper nigrum* (black pepper) and *Piper longum* (long pepper). Piperine is not only present as a seasoning but also in various preparations of traditional medicine, including the oldest medical science, practiced in India since antiquity (Ayurveda) [4]. The molecular structure of piperine is composed by two different groups that are electronically conjugated: a methylenedioxybenzene and an oxopentadienyl chromophore [5], leading to characteristic ultraviolet (UV) absorptions at 244, 255, 310 and 342 nm (Figure S1). Piperine has been widely studied by the scientific community because of its anti-inflammatory, anti-carcinogenic, immunomodulatory and hepatoprotective activities [6].

Despite the investigations reported in the literature, showing promising results of piperine as an inhibitor of inflammatory response via downregulation of the IL-1β pathway, the characterisation of molecular interactions is still unknown. In general, the discovery and development of a new drug is an expensive and time-consuming process, where the therapeutic effects and hazards to health are analysed by carrying out series of experimental and in vivo tests [7]. Nevertheless, in recent years, alternative methods have been developed to reduce the requirement of animals in the experiments and, as an alternative, the drug discovery field has moved toward more rational strategies based on protein–ligand interactions [7].

Considering the pharmacological importance of IL-1β and the lack of information about the interaction with piperine, the aim of this work was to characterise the IL-1β-piperine complex by means of experimental and computational biophysical assays.

To reach such a goal, time-resolved and steady-state fluorescence experiments were carried out to determine the quenching mechanism, the binding constant, number of binding sites and thermodynamic parameters. Circular dichroism (CD) spectroscopy was applied to determine the IL-1β secondary structure fractions and to elucidate possible changes in the secondary structure of the protein under either the effect of temperature or due to interaction with piperine. With the use of molecular docking, the binding site and the microenvironment of interaction were elucidated. In addition, biased molecular dynamics were performed to obtain the theoretical free energy of the binding through the calculation of potential mean force (PMF). The conformations between the bound and unbound states were explored with the support of the umbrella sampling method.

## 2. Materials and Methods

### 2.1. Reagents

Piperine (>97%) was purchased from Sigma-Aldrich Chemical Co. (Schnelldorf, Bavaria, Germany), as dibasic sodium phosphate (>99%) reagents, anhydrous citric acid (>99%), and sodium chloride (>99%). Lyophilised IL-1β (>97%) was purchased from GenScript Biotech (Piscataway, NJ, USA). Methanol alcohol was purchased from Dynamics Química Contemporânea LTDA (Indaiatuba, SP, Brazil). All the materials purchased were used as supplied. Ultrapure water was prepared by a Millipore water purification system—Direct-Q UV-3 (Merck KGaA, Darmstadt, Germany). Lyophilized IL-1β was reconstituted in a 50-mM phosphate buffer containing 150-mM sodium chloride, and the pH was adjusted to 7.4 with anhydrous citric acid. Stock solutions of piperine were prepared in pure methanol. The concentrations of piperine and IL-1β solutions were determined by UV-VIS experiments performed on Biospectro spectrophotometer (Biospectro, Curitiba, PR, Brazil), using the extinction coefficient at 16,500 M^−1^cm^−1^ at 345 nm for piperine and 11,460 M^−1^cm^−1^ at 280 nm for IL-1β.

### 2.2. Steady-State Fluorescence Spectroscopy

Fluorescence experiments were performed on the Lumina (Thermo Fisher Scientific, Waltham, MA, USA) stationary state spectrofluorimeter equipped with a thermal bath and Xenon lamp. A 100-μL quartz cuvette with a 10 × 2 mm optical path was used in the experiments. The widths of the excitation and the emission slits were adjusted to 10 nm. The wavelength of 295 nm was used to excite the single tryptophan residue of IL-1β (Trp120). The emission spectra were obtained in the range from 305 to 570 nm with a resolution of 1.0 ± 5.0 nm. Each emission point collected was the average of 15 accumulations. The software ScanWave was used to collect the measured data.

In the binding equilibrium experiments, aliquots of piperine (increment of 4 μM) were added in IL-1β solution at 4 μM. Measurements were performed at 288, 298, and 308 K. In the interaction density function analysis, small aliquots of piperine (increments of 1 μM) were added to IL-1β solutions at 4 μM, and 8 μM at a fixed temperature (298 K). In all experiments, the final volume of methanol in the buffer was less than 1.0%.

The correction of the inner filter effects was done with Equation (1), where *F_corr_* and *F_obs_* are corrected and observed fluorescence intensities, and *A_ex_* and *A_em_* are the absorbance at the excitation and the emission wavelengths, respectively, considering a cuvette of 10 × 10 mm of optical path [8].

The Stern–Volmer constant (K_SV_) and bimolecular constant (k_q_) were obtained from Equation (2)

(1) $$F_{corr}=\;F_{obs}\cdot \;10^{\frac{\left(5.A_{ex}+\;A_{em}\right)}{10}}$$

(2) $$\frac{F_{0}}{F}=1+K_{\mathrm{SV}}\cdot \left[\mathrm{piperine}\right]=1+{\;k}_{q}\cdot \tau_{0}\cdot \left[\mathrm{piperine}\right]$$

where F is the observed fluorescence intensity, F_0_ is the fluorescence intensity in the absence of piperine and τ_0_ is the Trp120 lifetime in the absence of piperine.

### 2.3. Time-Resolved Fluorescence

Fluorescence lifetime measurements were performed using a Mini-tau filter-based fluorescence lifetime spectrometer coupled to a Time-Correlated Single Photon Counting (TCSPC) system (Edinburgh Instruments, Livingston, UK). Aliquots of piperine were added in the IL-1β solution at 10 μM. Piperine concentration varied from 0 to 49 μM. Experiments were carried out at 298 K.

The sample was excited at 295 nm using a picosecond pulsed light emitting diode (LED), and fluorescence decay was collected using a 340 nm filter. The fluorescence decay profile (Figure S2) was fitted using multiexponential decay (Equation (3)), where τ_i_ is the lifetime of each component, and α_i_ is the contribution of each component to total fluorescence decay. The average lifetime <τ_avg_> was calculated using Equation (4) (Table S1).

(3) $$I_{T}=\;\overset{n}{\underset{i=1}{\sum }}\alpha_{i}\cdot e^{\frac{-T}{\tau_{i}}}$$

(4) $${\;\tau }_{\mathrm{avg}}=\frac{\alpha_{1}\tau_{1}{}^{2}+\alpha_{2}\tau_{2}{}^{2}}{\alpha_{1}\tau_{1}+\alpha_{2}\tau_{2}}$$

### 2.4. Circular Dichroism Spectroscopy

Circular dichroism spectra were recorded at 288, 298, and 308 K on a Jasco J-815 spectropolarimeter model DRC-H (Jasco, Easton, MD, USA) equipped with a demountable quartz cell with a 0.01 cm optical path length. The CD spectra were recorded from the 200 to 260 nm range with a scan rate of 20 nm/min and a spectral resolution of 0.1 nm. For each spectrum, 15 accumulations were performed. The molar ratios of IL-1β and piperine were 1:0 and 1:12, and the buffer spectrum was subtracted. The ellipticity *θ* collected in millidegrees was converted to mean residue ellipticity [*θ*] (deg cm^2^ dmol^−1^) using Equation (5).

(5) $$\left[\theta \right]=\frac{\theta \left(\mathrm{mdeg}\right)}{10.\left[P\right].l.n}$$

The secondary structures percentages were calculated with CDPro applying the CONTIN method with the SP43 protein library [9].

### 2.5. Molecular Docking

The alkaloid structure parameters used in molecular docking were the same as those obtained from ab initio calculations of the previous work [10] and the structure of the protein was obtained from chain A of PDB-1ITB. AutoDockTools [11] software of the MGL program Tools 1.5.4 was used to prepare the proteins by adding polar hydrogen atoms and Gasteiger charges. Blind docking was performed to explore the whole IL-1β protein following the previously described procedures [12]. The maps were generated by AutoGrid 4.2 program with a spacing of 0.4583 Å, dimension of 126 × 126 × 108 points and grid center coordinates of 41.028, −0.369 and 12.346 for x, y and z coordinates, respectively. The AutoDock 4.2 [11] was used to investigate the protein binding site using the Lamarckian Genetic Algorithm (LGA) with a population size of 150, maximum number of generations of 27,000 and energy evaluations equal to 2.5 × 10^6^. The other parameters were selected as the software default. To generate different conformations, the total numbers of runs was set to 100. The final conformation was chosen among the most negative energy that belong to the most representative cluster (Figure S3) and visualized by visual molecular dynamics software (VMD) [13]. The binding microenvironment was generated by LigPlot [14].

### 2.6. Molecular Dynamics

The simulations of the complex IL-1β/piperine were performed with GROMOS54a6 force field [15] by Gromacs v.5.1.4 [16]. The complex was placed in a rectangular box, solvated with the simple point charge water (SPC) [17] and neutralized with NaCl in a concentration of 150 mM. The energy minimization was performed with the steepest descent. The first step of equilibration was performed in an NVT ensemble for 100 ps. The system was coupled to the V-rescale thermostat [18] at 298 K. All bonds were constrained with the LINCS algorithm [19], the cut-off for short-range non-bonded interactions was set at 1.4 nm and long-range electrostatics were calculated using the Particle Mesh Ewald (PME) algorithm [20]. The second step of equilibration was performed in the NPT ensemble coupled to Parrinello-Raman barostat [21] to isotopically regulate the pressure for 100 ps. The pulling of piperine from IL-1β pocket was performed without restraints to allow the protein conformational changes. The reaction coordinate ξ was chosen as the distance between the Glu111 carbon atom (CA index 1129) and piperine carbon atom (CAE 1585) (Figure S4). Piperine was pulled away from IL-1β binding site in Z direction until the reaction coordinate reached 6 nm (Figure S5), using a spring constant of 800 kJ/mol^−1^nm^−2^ and a pull rate of 0.01 nm/ns. The potential of mean force (PMF) profile [22] along the reaction coordinate was calculated with WHAM method [23]. Statistical errors were estimated with bootstrap analysis, with 1000 bootstraps properly autocorrelated.

## 3. Results and Discussion

### 3.1. Fluorescence Spectroscopy

At Figure 1, the quenching effect is shown, as monitored by the fluorescence spectra of Trp120 with the addition of piperine aliquots in a sample with fixed concentration of IL-1β. The centre of fluorescence band of IL-1β remained at 340 nm, revealing that the interaction of the protein with piperine did not change the polarity of the fluorophore (Trp120) microenvironment. A second fluorescence band was observed at 480 nm, due to piperine being excited at 295 nm. However, the full width half maximum (FWHM) of ± 25 nm for the band at 340 nm and ± 38 nm for the band at 480 nm guarantees that the bands did not overlap, allowing the fluorescence data to be handled accurately.

The mechanism of quenching can be defined as either static (ground state complex) or dynamic (diffusive encounters, collisions) process. The most common method reported in the literature to determine the quenching mechanism is the comparison of the ratio of fluorescence signals (F_0_/F) with the ratio of the lifetime values (τ_0_/τ). In the case of dynamic quenching, the system presents F_0_/F = τ_0_/τ; such behavior is not observed for static quenching [8,24]. Another method used to determine the quenching mechanism is the analysis of the bimolecular constant (k_q_ from Equation (8)). In general terms, for systems ruled by collisions (dynamic quenching), the bimolecular constant does not exceed the limit of 10^10^ M^−1^ s^−1^ [24].

**Figure 2:**
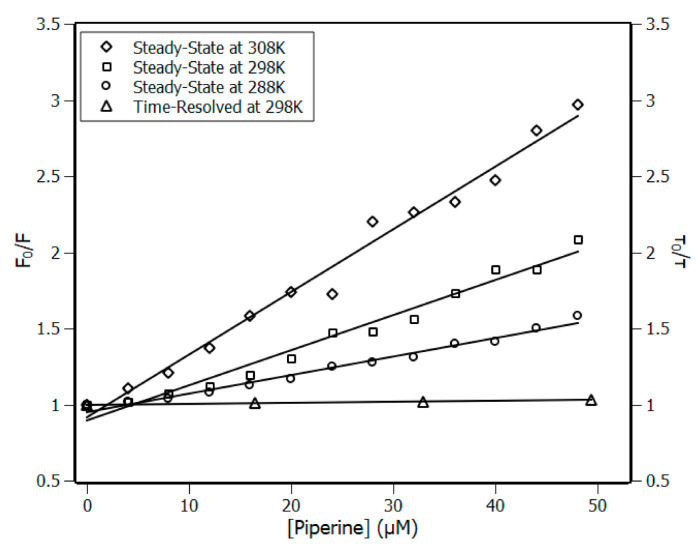
Left ordinate Stern–Volmer plots at three temperatures, 288, 298, and 308 K, and right ordinate time-resolved fluorescence lifetime plot at 298 K; [IL-1β] = 4 μM, [piperine] = 0–48 μM. Piperine increments of 4 μM. R^2^ > 0.98.

The Stern–Volmer plots at temperatures 288, 298 and 308 K, along with the lifetime plot at 298 K, are shown in Figure 2. The Stern–Volmer plots presented linear profiles, indicating that one quenching mechanism is predominant in the system [8]. In addition, the lifetime ratio (τ_0_/τ) revealed that the addition of piperine to the IL-1β solution did not influence the fluorescence lifetime of Trp120 significantly, once τ_0_/τ remained close to the unit. Figure 2 also showed the absence of equivalence between τ_0_/τ and F_0_/F, indicating that the quenching mechanism is static. To reinforce this result, the values of bimolecular constants (k_q_) were estimated with the order of magnitude of 10^12^ M^−1^ s^−1^ for the three temperatures analysed (Table 1); such values are two orders of magnitude greater than that allowed for collisional quenching [24]. All these results characterise the quenching mechanism as static, indicating that a complex has been formed by the IL-1β and piperine.

**Figure 3:**
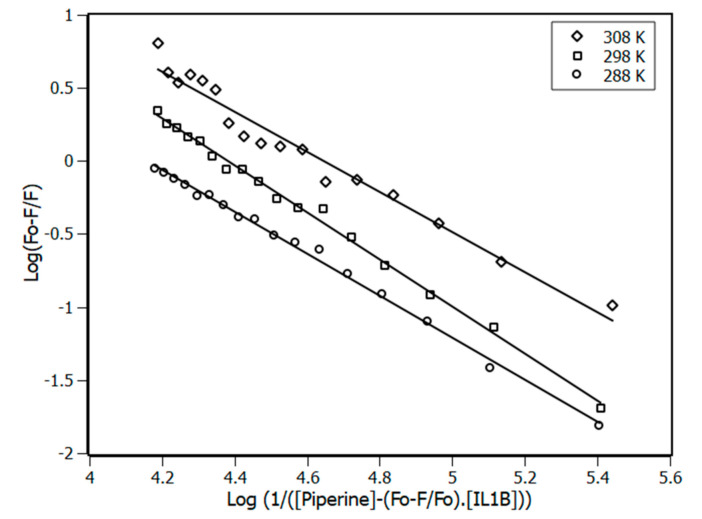
Double-log plots for the fluorescence quenching of IL-1β (4 μM) by the presence of piperine at 288, 298, and 308 K. R^2^ > 0.98.

The binding constant (K_a_) presented in Table 1 and the number of sites (n) were obtained from the plot at Figure 3 using the double-logarithm equation (Equation (6)).

(6) $$\log\left(\frac{F_{0}-F}{F}\right)=n.\log K_{a}-n.\log\left(\frac{1}{\left[\mathrm{piperine}\right]-\left(\frac{F_{0}-F}{F_{0}}\right)\cdot \left[\mathrm{IL}-1\beta \right]}\right)$$

The results revealed one binding site for piperine in the IL-1β, with the binding constant values increasing with the temperatures, as shown in Table 1.

### 3.2. Thermodynamic Analises

**Figure 4:**
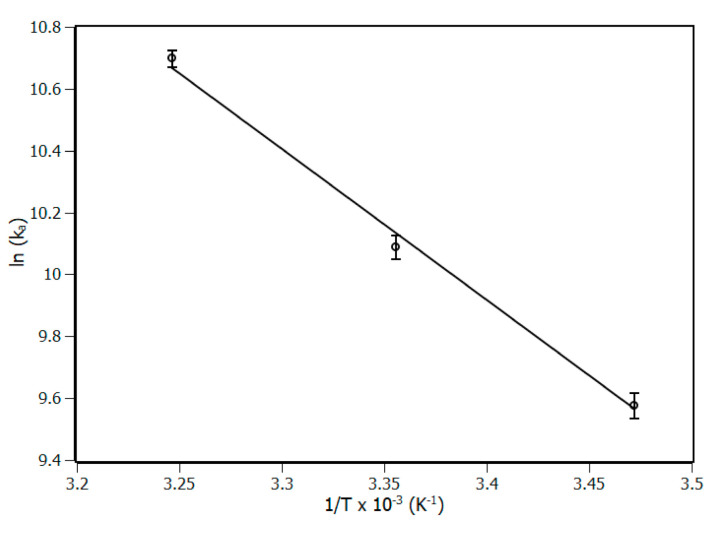
van’t Hoff plot for the complex IL-1β/piperine at 288, 298, and 308 K. R^2^ > 0.99.

The van’t Hoff equation (Equation (7)) was applied at the binding constant to obtain the thermodynamic parameters ∆S (entropy variation) and ∆H (enthalpy variation) (Figure 4), to describe the thermodynamic balance of the complex and to provide evidence of the molecular driving forces involved in the interaction [25].

(7) $$\ln K_{a}=-\frac{∆H}{R.T}+\frac{∆S}{R}$$

Gibbs free energy ∆G is another important thermodynamic parameter that can be obtained through Equation (8). ∆G exhibited negative values at the three temperatures, which indicated the spontaneity of the complex formation process [26]. According to the results of ∆S, ∆H and ∆G, resumed in Table 2, the values of ∆G were influenced by the change in temperature due to the high contribution of the entropic term (T.∆S). The fact that the system has exhibited T.∆S > 0 and T.∆S > ∆H is a strong evidence that forces of entropic nature, such as non-specific interactions, were playing a major role in the formation and stabilization of the complex [26,27].

(8) ∆G=∆H−T.∆S

### 3.3. Interaction Density Function (IDF)

**Figure 5:**
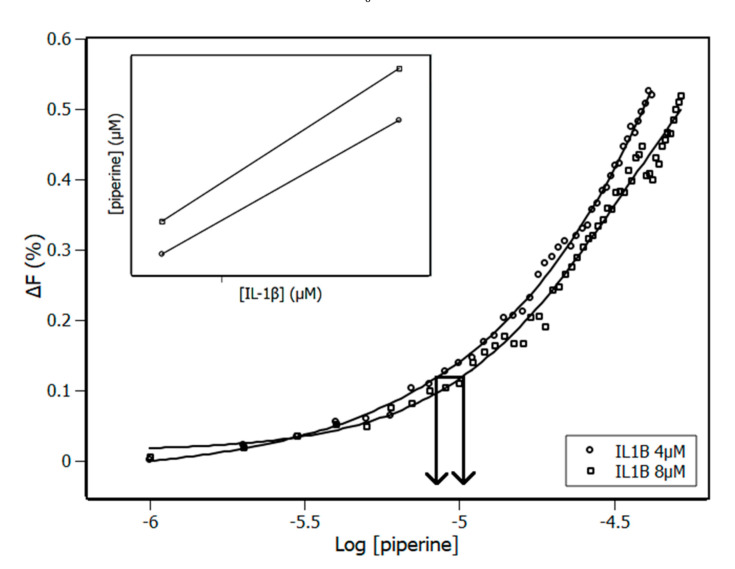
Plot of ∆F versus log [piperine] obtained from piperine titration experiments with IL-1β concentrations of 4 and 8 μM at 298 K. The horizontal solid line indicates the same percentage of quenching at different IL-1β concentrations. The inset shows an example of the lines obtained for each set of quenching percentage.

The IDF method was applied in this complex as a second method to obtain an in-depth description of the binding site of IL-1β. It is a methodology used to treat experimental data, but differently from the binding equilibrium model, IDF does not make use of any model a priori; instead, it makes use of the mass conservation law [28]. Because of that, the advantage of applying IDF is the possibility of not only determining the number of binding sites but also identifying possible cooperativity occurrence. IDF considers that, if the free ligand concentration ([piperine]_free_) is the same at two or more different concentrations of total protein ([IL-1β]), the average interaction density (Συi) will also be the same, and consequently the system will have the same variation in the percentage of quenching (ΔF). The percentage of fluorescence quenching is given by Equation (9), where F is the observed fluorescence signal of IL-1β at the presence of piperine and F_0_ without piperine. Figure 5 shows the plot of ΔF versus log [piperine] for two known concentrations of IL-1β, both adjusted by a sigmoidal function.

(9) $$∆F=\frac{\left|F-F_{0}\right|}{F_{0}}\;\cdot \;100\%$$

Free ligand concentration [piperine]_free_ and the average of interaction density (Συi) are related to each other through the expression of mass conservation (Equation (10)).

(10) $$\left[\mathrm{piperine}\right]={\left[\mathrm{piperine}\right]}_{\mathrm{free}}+\;\left(\sum \nu_{i}\right)\cdot \left[\mathrm{IL}-1\beta \right]$$

By means of the plot showed on Figure 5, the values of [IL-1β] and [piperine] for each ΔF were obtained. The inset of Figure 5 shows the plot of [piperine] versus [IL-1β] for each ΔF, in which Σν_i_ was obtained from the slope and [piperine]_free_ was obtained from the y-intercept of the linear function.

**Figure 6:**
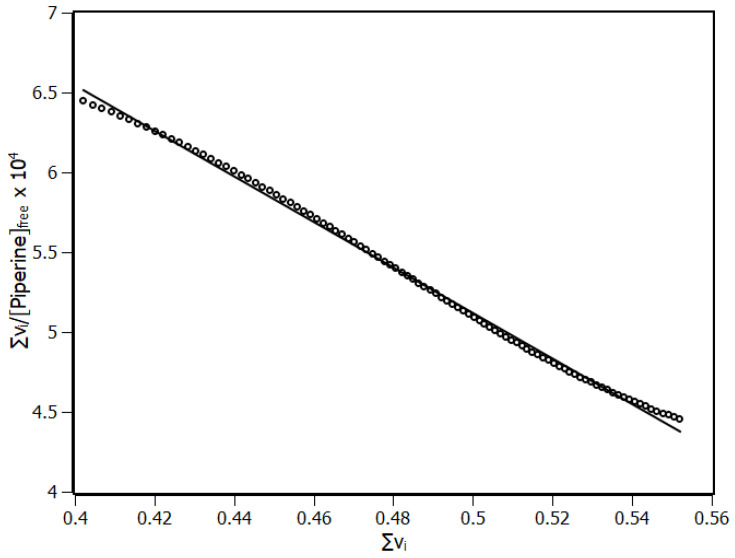
Scatchard plot of IL-1β and piperine at 298 K temperature with linear regression (black line). R^2^ > 0.99.

According to the IDF results, the Scatchard plot was built (Figure 6). This plot presented a unique linear profile, indicating that all IL-1β binding sites for piperine are equivalent and independent, exhibiting a non-cooperative state [29]. The number of sites (n) and the binding constant (K_b_) were obtained through linear regression based on Equation (11).

(11) $$\frac{\sum \nu_{i}}{{\left[\mathrm{piperine}\right]}_{\mathrm{free}}}=n.K_{b}-{\;K}_{b}\cdot \sum \nu_{i}$$

The IDF method with the Scatchard plot revealed one binding site with binding constant K_b_ of (14.3 ± 0.1) × 10^4^ M^−1^. The IDF and binding equilibrium methods agreed on the number of binding sites and differed somewhat in terms of binding constant. In general, the difference observed in the values of binding constant calculated through IDF and binding equilibrium is due to the fact that the binding equilibrium method uses a first reaction model to determine the constant and as a consequence, the binding constant is underestimated. Similar results were reported and discussed previously in the literature [10,27].

### 3.4. Circular Dichroism (CD)

**Figure 7:**
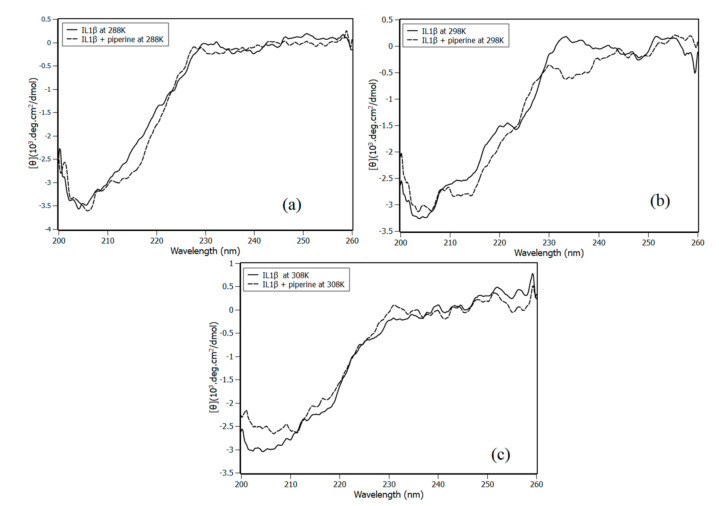
IL-1β circular dichroism experiments in the presence and absence of piperine with 1:12 stoichiometry at (**a**) 288 K, (**b**) 298 K and (**c**) 308 K.

Circular dichroism experiments were performed to distinguish the possible influence of temperature and piperine in the secondary structure of IL-1β (Figure 7).

The CD IL-1β spectra were deconvolved and analysed with the CDpro software using the CONTINNL algorithm and the SP43 library with 43 soluble proteins. The results indicated that the structure of IL-1β is predominantly composed of beta sheets (~39%); a similar CD profile was obtained for murine and human IL-1β in previous work [30,31].

The deconvolution of the spectra obtained in temperature range from 288 to 308 K showed slight changes in secondary structures (Table 3). Circular dichroism results also indicated that the interaction with piperine did not cause significant changes in IL-1β structure (2%). Although this 2% of difference on the secondary structures may be statistically low, this does not guarantee that is biochemically insignificant.

### 3.5. Computational Modeling

**Figure 8:**
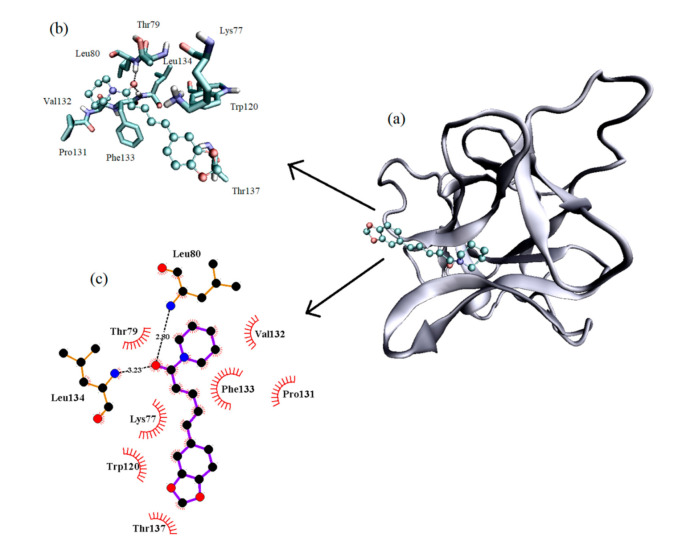
IL-1β binding site of piperine predicted by molecular docking in (**a**) a general view, (**b**) with the amino acids (licorice outfit) that compose the binding environment; hydrogen bonds are highlighted with dots and (**c**) the interactions represented by LigPlot.

Molecular docking was performed in order to disclose the binding site of piperine in the protein. According to molecular docking results (Figure 8), piperine binds to the protein with the energy score of −6.08 kcal/mol. The binding site found is basically composed of non-polar amino acids such as Leu80, Leu134, Val32, Phe133, Pro131 and Trp120, with limited polar amino acids (charged or not) such as Lys77, Thr137 and Thr79. Besides that, piperine performs two hydrogen bonds with the amino acids Leu80 and Leu134 with bond lengths of 2.90 and 3.23 Å, respectively.

**Figure 9:**
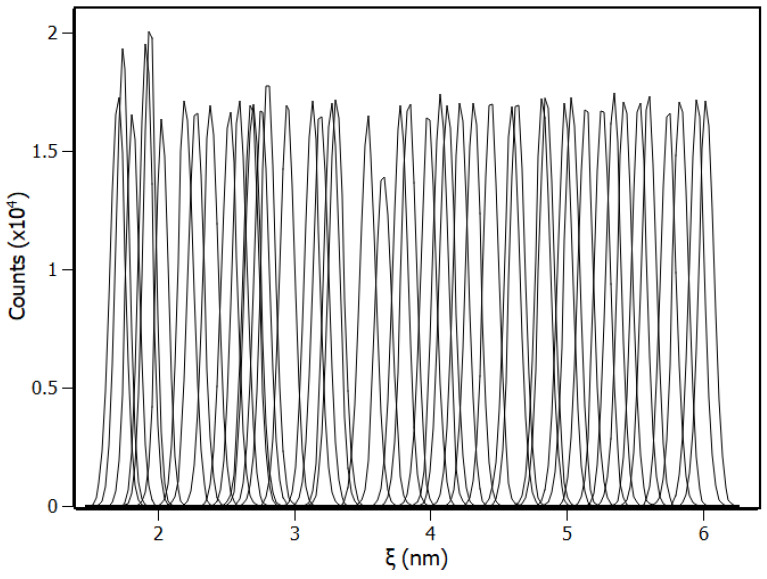
Configuration histograms of the pulling in *z*-axis from ξ = 1.7 nm to ξ = 6 nm with the windows distance as being 0.1 nm.

To confirm the binding site predicted by molecular docking, the dissociation process was simulated through biased molecular dynamics, and the configurations obtained between the bound and unbound states were used for umbrella sampling. The theoretical binding energy of the complex (∆G_pred_) was calculated by means of the potential mean force (PMF) and compared with the experimental binding free energy ∆G obtained from the thermodynamic parameters (see Section 3.2). During the pulling simulation, piperine was pulled from the protein in the z direction until the energy remained stable, which indicated that the ligand dissociated from the IL-1β and the intermolecular interactions between the piperine and the IL-1β were not significant anymore. To assure a high sampling of the system and that most of the system conformations were accessed, the system was sampled with windows of 0.1 nm of distance between each configuration (Figure 9). According to Figure 9, the high sampling was verified by the overlapping of the histograms of the configuration for each umbrella window.

**Figure 10:**
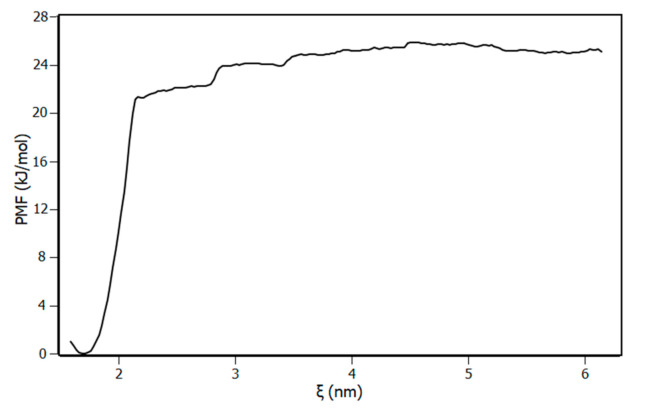
Potential of mean force (PMF) for the dissociation of piperine from IL-1β. Piperine was pulled away from the protein using the reaction coordinate ξ as being the distance between Glu111 carbon atom (CA index 1129) and piperine carbon atom (CAE 1585) with the force applied in Z direction (see Figure S4).

According to the PMF results (Figure 10), the predicted binding free energy at 298 K was (−25.6 ± 1.2) kJ/mol, corroborating with the experimental binding free energy determined by the van’t Hoff analysis (−25.09 ± 0.09) kJ/mol (Table 2). This result confirms the binding site disclosed by molecular docking.

**Figure 11:**
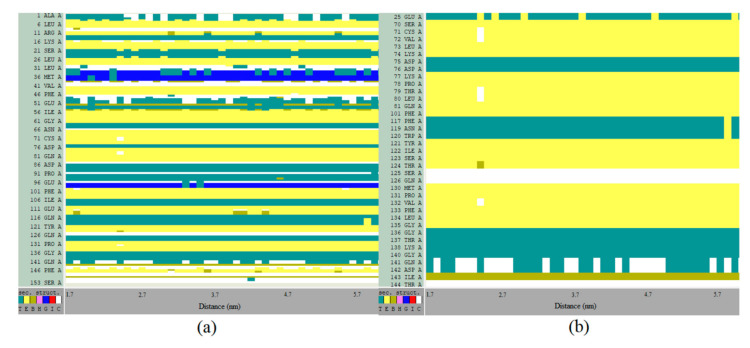
IL-1β secondary structures changes of (**a**) the whole protein and (**b**) only the binding site during the dissociation of piperine (from ξ = 1.7 nm to ξ = 5.7 nm), where T represents the Turns (green), E the β-sheet (yellow), B the isolated bridge(brown), H the alpha-helix (pink), G the 3–10 helix (blue), I the Pi-helix (red) and C the coil (white).

The stability of the secondary structures was inquired during the dissociation of piperine from IL-1β (Figure 11). According to the results, from the initial moment (ξ = 1.7 nm) when piperine was in the binding site to the final moment that the complex was dissociated (ξ = 6.0 nm), the protein had its secondary structure preserved both for the whole protein structure (Figure 11a) and binding site (Figure 11b). Such results correspond to the circular dichroism spectra that revealed that, during the interaction with piperine, the IL-1β structure was preserved.

## 4. Conclusions

In the present work, the interaction between piperine and the cytokine IL-1β was investigated by means of experimental and computational molecular biophysical tools. Steady-state and time-resolved fluorescence showed that IL-1β at the presence of piperine presented a static-quenching process, which means that a complex was formed. Fluorescence spectroscopy analysis revealed that IL-1β has one binding site for piperine, with the binding constant being (14.3 ± 0.1) × 10^4^ M^−1^, while the thermodynamic parameters indicated that the complexation was a spontaneous process (ΔG = −25.09 kJ/mol) with high entropic contribution, indicating that non-specific interactions played an important role in both the complex formation and stabilization. Circular Dichroism results showed that the interactions of piperine with the IL-1β did not cause significant secondary structure changes. The binding site was unveiled by molecular docking as being composed mainly by non-polar amino acids that are characteristic of non-specific interactions. Potential mean force profile calculated from the umbrella sampling simulations lead to a ∆G_pred_ of (25.6 ± 1.2) kJ/mol, which is in concurrence with the free energy calculated from van’t Hoff analysis (25.09 ± 0.09) kJ/mol. Molecular dynamics simulation showed that the protein secondary structures remained preserved during the pulling of piperine, with fluctuations similar to CD spectra. A multispectroscopic evaluation aided by molecular docking and dynamic elucidated, in detail, IL-1β/piperine molecular interaction, which can support further drug discovery studies.

## Figures and Tables

**Table 1:** Stern–Volmer constant (K_SV_), bimolecular constants (k_q_) and binding constant (K_a_) for the complex IL-1β and piperine at 288, 298 and 308K.

Temperature (K)	Stern-Volmer (K_SV_) × 10^4^ M^−1^	Bimolecular (K_q_) × 10^12^ M^−1^ s^−1^	Binding (K_a_) × 10^4^ M^−1^
288	1.22 ± 0.05	2.54 ± 0.01	1.44 ± 0.06
298	2.32 ± 0.12	4.87 ± 0.01	2.41 ± 0.09
308	4.13 ± 0.16	8.59 ± 0.01	4.42 ± 0.12

**Table 2:** Thermodynamic parameters of the IL-1β/piperine complex at temperatures of 288, 298 and 308 K.

T (K)	∆G (kJ/mol)	∆H (kJ/mol)	T.∆S (kJ/mol)
288	−22.87 ± 0.10	41.26 ± 2.85	64.13 ± 2.88
298	−25.09 ± 0.09	41.26 ± 2.85	66.36 ± 2.88
308	−27.32 ± 0.07	41.26 ± 2.85	68.58 ± 2.88

**Table 3:** Main composition of secondary structures of pure IL-1β and with piperine at the stoichiometry protein:piperine (1:12) at 288, 298 and 308 K.

IL-1β	α-Helices (%)	β-Sheet (%)	Turns (%)	Coil (%)
Pure at 288 K	4	39	22	35
Pure at 298 K	5	38	22	35
Pure at 308 K	4	39	23	34
+ piperine 288 K	4	41	22	34
+ piperine 298 K	5	40	23	32
+ piperine 308 K	4	40	23	34

## Supplementary Materials

The following are available online at https://www.mdpi.com/2218-273X/10/9/1337/s1, Figure S1: Piperine at 10 μM absorption spectrum at dibasic sodium phosphate in pH = 7.4 at 298 K. Cell length = 1 cm: title, Figure S2: Time-resolved fluorescence decay of (a) IL-1β with Piperine (→e) from 12 to 49 μM. [IL-1β] = 10 μM, T = 298 K and λex = 295 nm, Figure S3: Molecular docking clusters with their respectives energy scores, Figure S4: The atoms picked to define the reaction coordinate (ξ). For the protein was chosen the atom CA with index 1129 from the amino acid Glu111. For piperine the atom chosen was CAE with index of 1585, Figure S5: Pulling profile during the pulling simulation. *Y*-axis is the value of reaction coordinate (ξ) and *x*-axis is the time of simulation. Table S1: Tryptophan lifetime in different stoichiometries IL-1β: Piperine.
