# Supplementary material for: The Cytokine IL-1β and Piperine Complex Surveyed by Experimental and Computational Molecular Biophysics
Source: Biomolecules. 2020 Sep 18;10(9):1337. doi: 10.3390/biom10091337 (PMC7563551; doi:10.3390/biom10091337)
Supplement: Supplementary file 1 [file biomolecules-10-01337-s001.pdf]

biomolecules

Supplementary material

The cytokine IL-1β and piperine complex surveyed by experimental and computational molecular biophysics.

Gabriel Zazeri[1], Ana Paula Ribeiro Povinelli[1], Marcelo de Freitas Lima[2], Marinônio Lopes Cornélio[1,*]

[1] Departamento de Física, Instituto de Biociências, Letras e Ciências Exatas (IBILCE), UNESP, Rua Cristovão Colombo 2265, CEP 15054-000, São José do Rio Preto, SP, Brazil. (G.Z) gabriel.zazeri@unesp.br, (A.P.R.P) ana.povinelli@unesp.br.

[2] Departamento de Química, Instituto de Biociências, Letras e Ciências Exatas (IBILCE), UNESP, Rua Cristovão Colombo 2265, CEP 15054-000, São José do Rio Preto, SP, Brazil. (M.d.F.L) marcelo.f.lima@unesp.br

* Correspondence: m.cornelio@unesp.br

Received: date; Accepted: date; Published: date

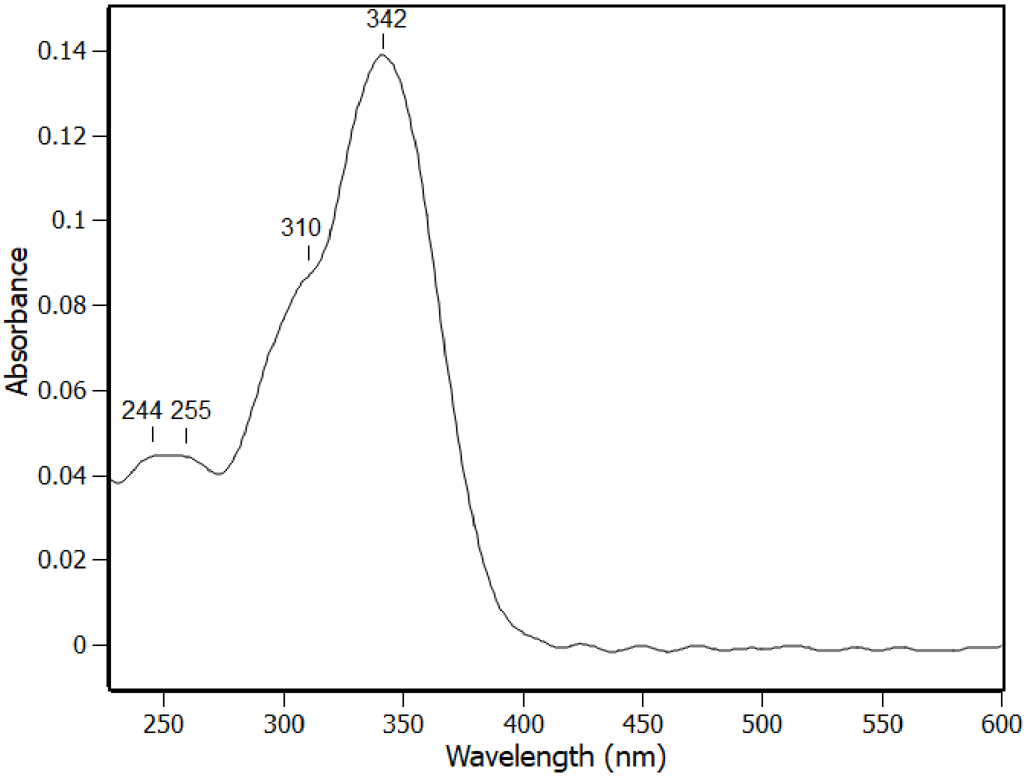

Figure S1: Piperine at 10μM absorption spectrum at dibasic sodium phosphate in pH=7.4 at 298K. Cell length = 1cm

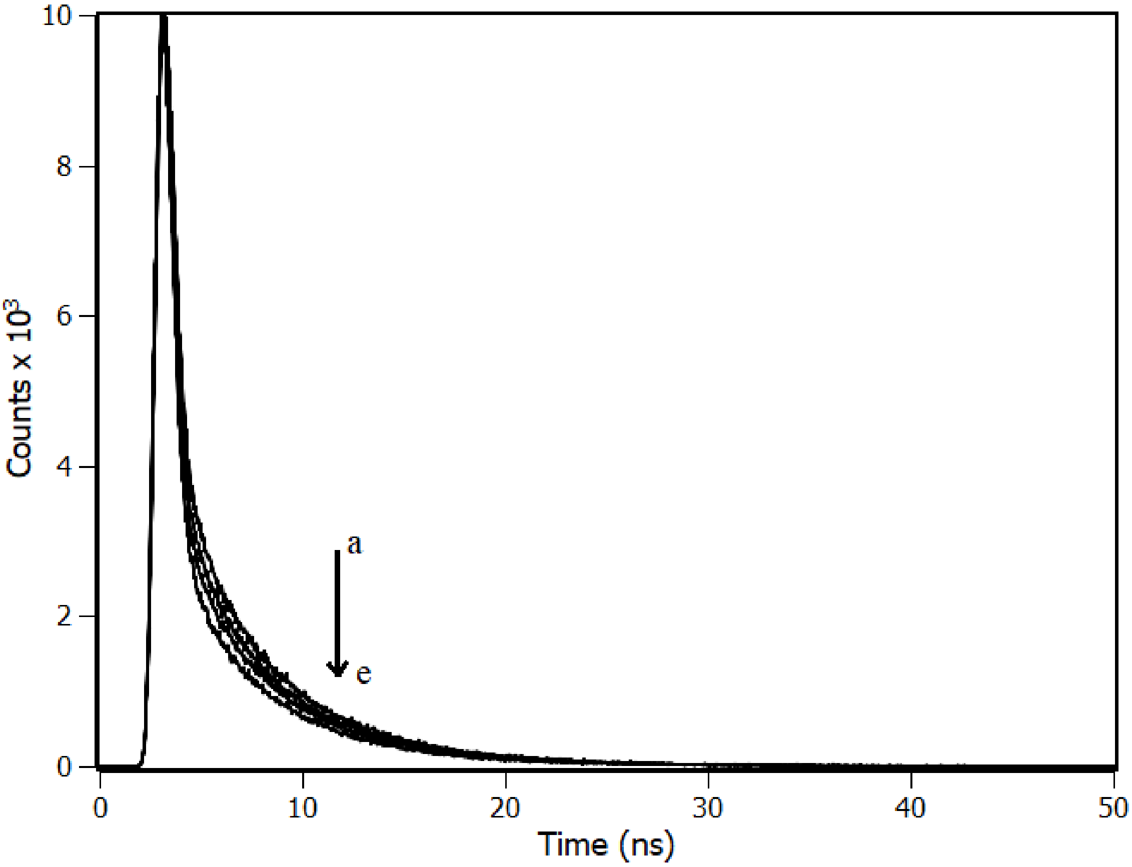

Figure S2: Time-resolved fluorescence decay of (a) IL-1β with Piperine (→e) from 12 to 49μM. [IL-1β] = 10μM, T = 298K and λ_{ex} = 295nm.

Table S1: Tryptophan lifetime in different stoichiometries IL-1β:Piperine.

[piperine](μM)	α_1	τ_1 (ns)	α_2	τ_2 (ns)	τ_{avg} (ns)
0	0.20	0.80	0.66	4.95	4.81
16	0.22	0.79	0.61	4.9	4.76
32	0.26	0.74	0.64	4.92	4.73
49	0.29	0.71	0.62	4.88	4.67

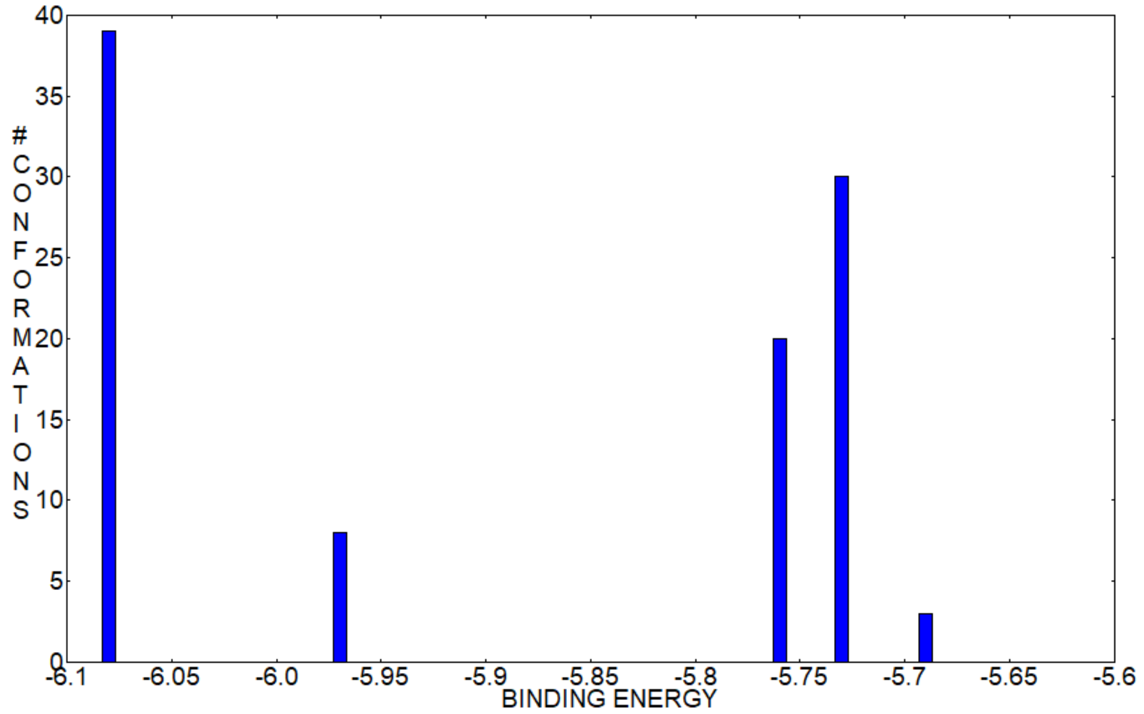

Figure S3: Molecular docking clusters with their respectives energy scores

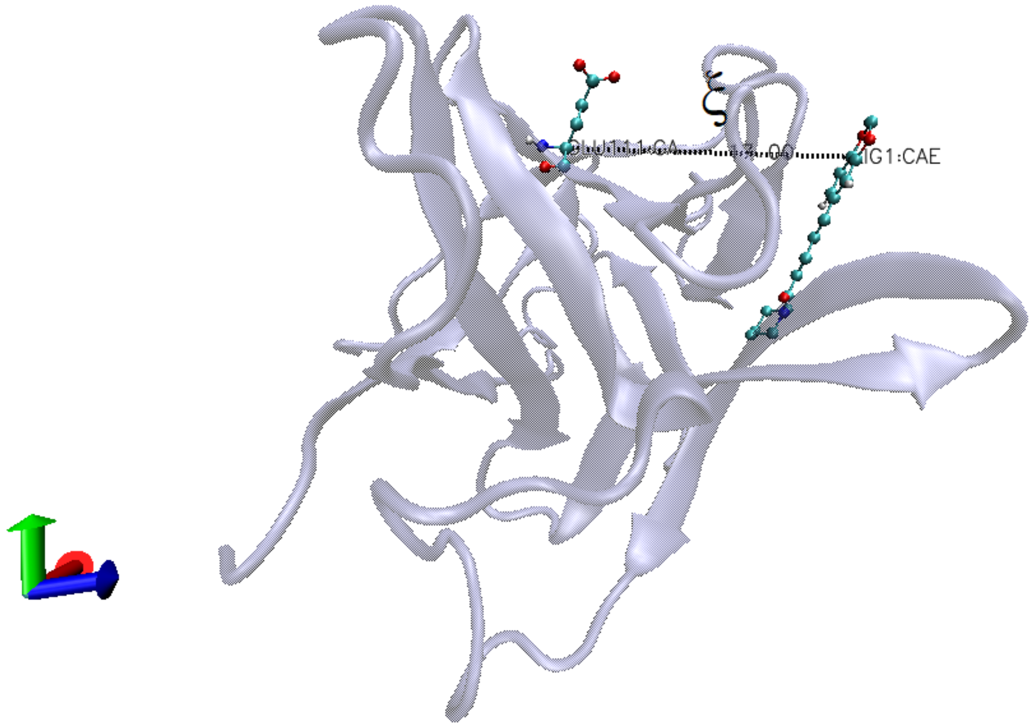

Figure S4: The atoms picked to define the reaction coordinate (ξ). For the protein was chosen the atom CA with index 1129 from the amino acid Glu111. For piperine the atom chosen was CAE with index of 1585.

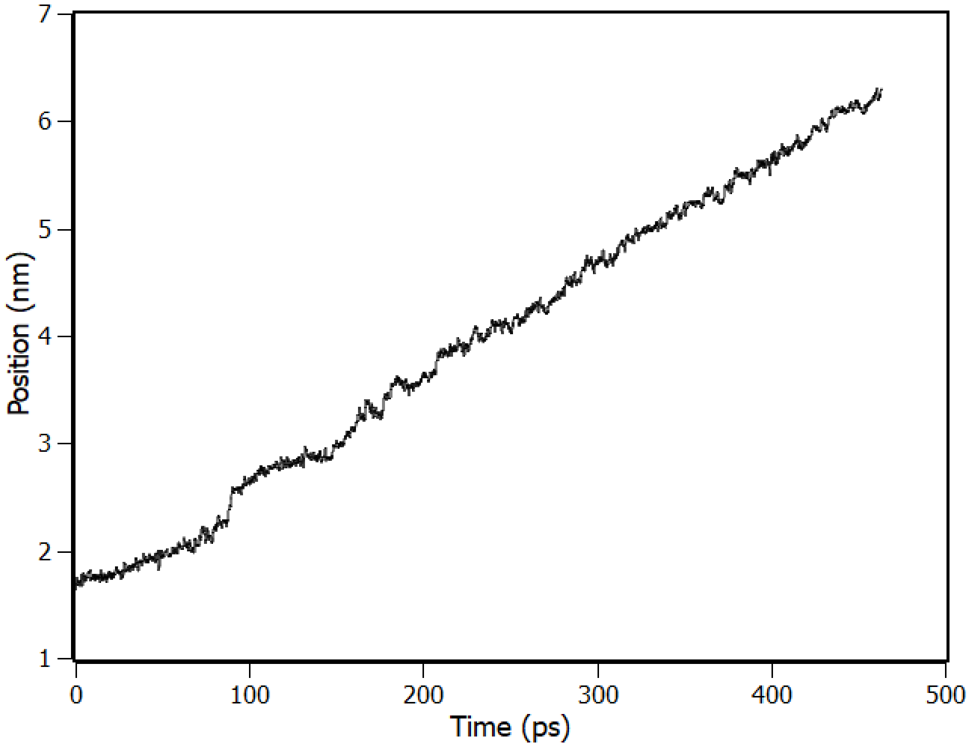

Figure S5: Pulling profile during the pulling simulation. Y-axis is the value of reaction coordinate (ξ) and x-axis is the time of simulation.

© 2020 by the authors. Submitted for possible open access publication under the terms and conditions of the Creative Commons Attribution (CC BY) license (http://creativecommons.org/licenses/by/4.0/).
